# Dupilumab, corticosteroids and their combination for the treatment of bullous pemphigoid^☆^

**DOI:** 10.1016/j.abd.2024.04.012

**Published:** 2024-12-17

**Authors:** Guirong Liang, Hua Qian, Chao Sun, Hanmei Zhang, Zhiliang Li, Suo Li, Ke Jing, Chenjing Zhao, Yuan Wang, Ruiyu Xiang, Xiaoguang Li, Suying Feng

**Affiliations:** a Hospital for Skin Diseases, Institute of Dermatology, Chinese Academy of Medical Sciences & Peking Union Medical College, Nanjing, China; b School of Public Health and Laboratory Medicine, Hunan University of Medicine, Huaihua, Hunan, China; c Department of Laboratory Medicine, Medical College, Dalian University, Dalian, Liaoning, China; d Department of Dermatology, Central Hospital Affiliated to Shandong First Medical University, Jinan, China; e Department of Dermatology, The Second Affiliated Hospital of Kunming Medical University, Kunming, China

**Keywords:** Corticosteroids, Dermatosis bullosa IgA lineal, Dupilumab, Pemphigoid, bullous, Treatment outcome

## Abstract

**Background:**

Conventional systemic corticosteroid therapy for bullous pemphigoid (BP) has been challenged due to severe adverse events. Dupilumab has emerged as an alternative therapeutical option of BP patients.

**Objectives:**

To evaluate the efficacy of dupilumab monotherapy and the combination with medium/low-dose corticosteroids for BP treatment.

**Methods:**

Thirteen, twenty-four and thirty-two BP patients treated with Dupilumab monotherapy (Dupi group), dupilumab combined with corticosteroids (Dupi + CS group), and corticosteroid monotherapy (CS group), respectively, were retrospectively analyzed for various clinical and laboratory parameters.

**Results:**

In the Dupi group, the total Bullous Pemphigoid Disease Area Index (BPDAI) Total, Erosion/Blister, Urticaria/Erythema and Itching NRS scores were all reduced significantly after 2–4 weeks of treatment, but the BPDAI Mucosal Score was not changed significantly at the end of the overextended time of treatment. All the above clinical parameters and many laboratory parameters (including the serum anti-BP180 autoantibodies [IgG] level, blood eosinophil count, and percentage) were significantly reduced in both Dupi + CS and CS groups after treatment, but no statistical differences were found in the reduction rates of these parameters between the two groups. However, the Dupi + CS group had less baseline dose and cumulative dosage of prednisone at the time of disease control, and fewer adverse effects were reported than the CS group.

**Study limitations:**

The retrospective design and small clinical sample size of the Dupi group.

**Conclusions:**

For BP patients, dupilumab monotherapy based on the treatment of atopic dermatitis can significantly improve skin lesions and pruritus symptoms but may be ineffective for oral mucosal lesions. The combination of dupilumab and medium/low-dose corticosteroids can achieve the same effect of corticosteroid therapy with superior safety.

## Introduction

Bullous pemphigoid (BP), the most common skin autoimmune subepidermal blistering disease, often occurs in the elderly, and typically manifests as tense bullae on edematous erythema, accompanied by severe itching.^1^, ^2^ BP autoantibodies mainly target two hemidesmosomal components, BP180 and BP230, and anti-BP180 autoantibodies have been recognized as the major pathogenic autoantibodies in BP.^1^ Corticosteroids and immunosuppressants are the two most common drugs for the treatment of BP,^3^ but long-term use of these drugs in the elderly is often accompanied by significant adverse reactions, such as hyperglycemia, osteoporosis and infection.^4^ Recent studies have found that the T-helper 2 cell (Th2) inflammatory response plays a role in the pathogenesis of BP.^5^, ^6^

The Th2-related cytokines IL-4 and IL-5, which are important for eosinophil chemoattraction, maturation, and functional activity, were upregulated in the skin lesions of BP patients and downregulated after corticosteroid treatment.^5^, ^7^, ^8^, ^9^ Meanwhile, eosinophils could participate in maintaining Th2-type reactions by producing IL-4, IL-5, and IL-13.^10^, ^11^ Dupilumab, a monoclonal antibody blocking IL-4 receptor α, can downregulate Th2 related signal pathway.^12^, ^13^ Dupilumab has been approved by the Food and Drug Administration (FDA) for the treatment of moderate to severe atopic dermatitis, and successful treatment of refractory BP by dupilumab has been reported in sporadic cases.^14^, ^15^, ^16^, ^17^, ^18^, ^19^, ^20^, ^21^, ^22^, ^23^, ^24^, ^25^, ^26^, ^27^, ^28^

In the present study, to further investigate the effectiveness of dupilumab on BP, the authors employed a total of 69 BP patients, including 13 patients treated with dupilumab monotherapy, 24 patients treated with dupilumab combined with corticosteroids and 32 patients treated with corticosteroids, to perform retrospective analyses by comparison of their therapy effects and changes of various clinical and laboratory parameters.

## Methods

### Patients and controls

Diagnostic criteria of BP: 1) Intensely pruritic blisters of the skin, 2) Positive linear IgG and/or C3c staining along the BMZ by direct immunofluorescence (DIF), 3) Positive IgG staining on the epidermal side of 1M-NaCl-split normal human skin by indirect immunofluorescence (ssIIF), 4) Positive IgG enzyme-linked immunosorbent assay (ELISA) of BP180 NC16A and/or BP230 (MBL, Nagoya, Japan, >9 U/mL). BP was diagnosed if the first criterion and at least two of the last three criteria were met. Exclusion criteria included drug-induced BP, patients with BP who were followed up for less than 3 months, and those who had used other biological agents within half a year before dupilumab treatment.

Sixty-nine BP patients diagnosed between June 2021 and June 2023 in the Hospital for Skin Diseases, Institute of Dermatology, Chinese Academy of Medical Sciences & Peking Union Medical College were involved in this study, including 13 patients treated with dupilumab monotherapy (dupilumab group, Dupi group), 24 patients treated with dupilumab combined with corticosteroids (dupilumab and corticosteroids group, Dupi + CS group), and 32 patients treated with monotherapy of corticosteroids (Corticosteroids group, CS group). In addition, all patients were initially prescribed 0.05% clobetasol propionate cream twice a day on the area of lesional skin and discontinued when the skin lesions had healed. In the present study, the main reasons of patients for dupilumab treatment include 1) Some patients cannot tolerate long-term or large-dose corticosteroids therapy due to underlying diseases (advanced age, hypertension, diabetes, kidney disease, hepatitis, tuberculosis, etc.); 2) Traditional treatment has proved ineffective; 3) Some patients refused to use corticosteroids therapy. Based on the regimen of dupilumab for the treatment of atopic dermatitis,^29^ patients in the Dupi group and Dupi + CS group first received 600 mg of dupilumab (induction dose) and then 300 mg every other week via subcutaneous injection.

In this study, all BP patients were followed up for 3–12 months. Whole blood samples were collected from all BP patients at baseline, 2–3 weeks after treatment, and 1–3 months after treatment. The Bullous Pemphigoid Disease Area Index (BPDAI) and the Numeric Rating Scale (NRS) score of itching were performed at baseline and 2–4 weeks after treatment. The BPDAI was determined by a panel of experts on BP and is composed of a score for the degree of erosions/blisters (range, 0–120 points), urticaria/erythema (range, 0–120 points) and mucosal erosions/blisters (range, 0–120 points), with higher scores indicating greater disease activity.^30^ BPDAI total score (range, 0–360 points) is the sum of three components including the BPDAI erosion/blister, BPDAI urticaria/erythema and BPDAI mucosal erosion/blister. Pruritus was evaluated by itching NRS score, which ranges from 0 (no itching) to 10 points (worst insupportable itching).^30^

At each follow-up, the occurrence of disease control, complete remission during tapering, relapse and adverse events were assessed. Disease control is defined as the point at which new lesions or pruritic symptoms cease to form and established lesions begin to heal.^30^ The disease control period is the period from baseline to disease control. Complete remission during tapering is defined as the absence of non-transient lesions while the patient is receiving more than minimal therapy.^30^ Relapse is defined as the appearance of 3 or more new lesions a month (blisters, eczematous lesions, or urticarial plaques) or at least one large (10 cm diameter) eczematous lesion or urticarial plaque that does not heal within 1-week, or the extension of established lesions or daily pruritus in a patient who has achieved disease control.^30^ Additionally, since the present study does not involve dosage reduction of Dupilumab, the concept of complete remission with medicine is exclusively used in the Dupi group, meaning the absence of non-transient lesions.

The changes of all clinical and laboratory parameters were evaluated by reduction rate, the percentage of decrease in the parameters after treatment compared to those before treatment.

The present study was approved by the Ethics Committee of the Institute of Dermatology and Hospital for Skin Diseases, Chinese Academy of Medical Sciences and Peking Union Medical College (2017-KY-022). Written informed consents were obtained from all involved patients and controls.

### DIF

For DIF, skin biopsy specimens of BP patients were embedded in the optimum cutting temperature compound. Cryosections (6 µm thick) of these specimens were incubated for 35-min at 37 °C with fluorescein isothiocyanate (FITC)-conjugated sheep anti-human IgG antibody (ZF0306, ZSGB-BIO, Beijing, China) or FITC conjugated rabbit anti-human C3c antibody (ZF0301, ZSGB-BIO, Beijing, China) both at a 1:100 dilution with Phosphate-Buffered Saline (PBS). The results were viewed independently using a fluorescent microscope (Eclipse 50i, Nikon, Tokyo, Japan) by at least two trained dermatologists.

### ssIIF

The skin tissues taken from normal individuals were washed with aseptic saline solution and then placed into a 50 mL sealed centrifuge tube with 1 moL/L NaCl (20°‒30 °C) for about 24 h, until the surface of the skin wrinkled, which indicated that the epidermal and dermal layers had been separated. This salt-split skin was next embedded with ornithine carbamyl transferase and cut into frozen sections 6 µm thick.^31^, ^32^ Serum samples of BP patients were diluted at 1:40 with PBS, and incubated with salt-split skin sections at 37 °C for 30-min. After thoroughly washing with PBS (10 mM, Ph 7.3), the sections were incubated with FITC-conjugated sheep anti-human IgG antibody (GTX26866, GeneTex, San Antonio, USA) at a 1:500 dilution with PBS at 37 °C for 30-min. The fluorescence intensity was assessed by at least two trained dermatologists.

### Elisa for detection of various IgG autoantibodies in patient sera

IgG antibodies against BP180 NC16A, BP230, Desmoglein 1 (Dsg1) and Dsg3 were measured by MESACUP BP180/BP230/Dsg1/Dsg3 TEST (MBL, Nagoya, Japan) according to the manufacturer's instruction. The serum samples were diluted to 1:101. The cut-off values for anti-BP180 NC16A, BP230, Dsg1 and Dsg3 antibodies (IgG) in serum samples were 9 U/mL, 9 U/mL, 20 U/mL and 20 U/mL, respectively.

### Routine blood test

The routine blood test of BP patients was measured using the colter cellular analysis system (UniCel DxH 600, Beckman Coulter, California, USA). Whole blood count and percentage for eosinophil were used for the analyses in the present study and the cut-off values were 0.02∼0.52*10^9^/L and 0.4∼8.0%, respectively.

### Total IgE

The level of serum total IgE, which was included in a screening panel of nutritive and inhalant allergens, was performed by Immuno CAP (Phadia/Thermofisher, Uppsala, Sweden). The cut-off value for total IgE in serum samples was 60 KU/L.

### Statistical analyses

Wilcoxon signed-rank test, Mann-Whitney *U* test, Kruskal-Wallis test and Fisher’s exact test was conducted depending on the type of analyses. The Steel-Dwass test was used for post-hoc pairwise comparisons between three subgroups after significant Kruskal-Wallis test. All data were presented as mean ± SD for descriptive purposes. The p-values were two-sided and performed with the appropriate statistical tests using GraphPad Prism software 9.4.0 and R software 4.1.2.^33^ A statistically significant difference was considered to be present at p < 0.05.

## Results

### Clinical and laboratory characteristics of BP patients

Sixty-nine BP patients were involved in this study, including 13 patients treated with dupilumab monotherapy (Dupi group), 24 patients treated with Dupilumab combined with Corticosteroids (Dupi + CS group), and 32 patients treated with monotherapy of Corticosteroids (CS group). The average age of BP patients in Dupi, Dupi + CS and CS groups were 73.2, 67.5 and 68.7 years old, respectively; and the ratio of male patients in these three groups was 61.5%, 58.3% and 40.6%, respectively. Clinical and immunological features of all patients at baseline were shown in Table 1.

**Table 1:** Clinical and laboratory characteristics of BP patients.

		Dupi group (n = 13)	Dupi + CS group (n = 24)	CS group (n = 32)
Age, year, mean ± SD		73.2 ± 19.2	67.5 ± 12.3	68.7 ± 15.4
Gender				
Male, n (%)		8 of 13 (61.5%)	14 of 24 (58.3%)	13 of 32 (40.6%)
Female, n (%)		5 of 13 (38.5%)	10 of 24 (41.7%)	19 of 32 (59.4%)
Duration of disease, months, mean ± SD		2.3 ± 1.4	4.7 ± 4.6	3.1 ± 3.3
Onset, n (%)				
Initial		12 of 13 (92.3%)	16 of 24 (66.7%)	24 of 32 (75.0%)
Relapse		1 of 13 (7.7%)	8 of 24 (33.3%)	8 of 32 (25.0%)
Involvement of oral mucous membrane		6 of 13 (46.2%)	8 of 24 (33.3%)	13 of 32 (40.6%)
DIF positive for linear IgG along the BMZ, n (%)	Pre	2 of 5 (40.0%)	11 of 15 (73.3%)	15 of 21 (71.4%)
DIF positive for linear C3c along the BMZ, n (%)	Pre	2 of 5 (40.0%)	9 of 15 (60.0%)	17 of 21 (81.0%)
ssIIF positive for IgG (epidermal side), n (%)	Pre	8 of 12 (66.7%)	16 of 21 (76.2%)	25 of 30 (83.3%)
Anti-Dsg1 autoantibodies (IgG) level, U/mL, mean ± SD	Pre	2.2 ± 1.8	3.7 ± 5.6	2.5 ± 4.0
Anti-Dsg3 autoantibodies (IgG) level, U/mL, mean ± SD	Pre	2.5 ± 1.9	2.4 ± 3.9	2.2 ± 3.5
Anti-BP180 autoantibodies (IgG) level, U/mL, mean ± SD	Pre	81.9 ± 49.2	113.9 ± 39.9	100.2 ± 49.7
	Post	82.1 ± 49.5 (n = 4)	78.9 ± 43.8 (n = 15)	66.3 ± 45.2 (n = 20)
	p-value	>0.999	**0.005**^a^	**<0.001**^a^
Anti-BP230 autoantibodies (IgG) level, U/mL, mean ± SD	Pre	51.8 ± 46.4 (n = 8)	45.6 ± 32.7 (n = 14)	62.3 ± 38.7 (n = 13)
	Post	31.7 ± 34.1 (n = 3)	30.6 ± 38.8 (n = 8)	34.1 ± 38.2 (n = 8)
	p-value	0.500	0.109	**0.008**^a^
Serum IgE level, KU/L, mean ± SD	Pre	1519.6 ± 1615.2	1463.5 ± 1841.1 (n = 23)	881.7 ± 1351.3 (n = 31)
	Post	1710.2 ± 220.2 (n = 5)	597.5 ± 666.9 (n = 9)	1359.5 ± 1856.1 (n = 13)
	p-value	0.062	0.652	0.622
Blood Eosinophil count,10^9^/L, mean ± SD	Pre	1.5 ± 1.8	1.6 ± 1.9	0.8 ± 0.8
	Post	3.2 ± 3.7 (n = 7)	0.2 ± 0.4 (n = 20)	0.1 ± 0.1 (n = 26)
	p-value	0.219	**<0.001**^a^	**<0.001**^a^
Blood Eosinophil percentage (%), mean ± SD	Pre	15.4 ± 15.6	10.8 ± 10.6	7.9 ± 7.3
	Post	23.1 ± 18.1 (n = 7)	1.6 ± 2.5 (n = 20)	0.7 ± 1.0 (n = 26)
	p-value	0.578	**<0.001**^a^	**<0.001**^a^
BPDAI Total Score, mean ± SD	Pre	48.1 ± 23.6	60.1 ± 23.7	51.1 ± 27.9
	Post	29.7 ± 18.1 (n = 10)	12.0 ± 10.0 (n = 13)	14.0 ± 22.8 (n = 18)
	p-value	**0.004**^a^	**<0.001**^a^	**<0.001**^a^
BPDAI Erosion/Blister Score, mean ± SD	Pre	28.9 ± 18.5	34.8 ± 19.8	26.7 ± 18.4
	Post	18.5 ± 12.8 (n = 10)	9.0 ± 9.9 (n = 13)	10.5 ± 15.6 (n = 18)
	p-value	**0.002**^a^	**<0.001**^a^	**<0.001**^a^
BPDAI Urticaria/Erythema Score, mean ± SD	Pre	16.9 ± 9.6	24.0 ± 11.8	22.7 ± 16.3
	Post	4.9 ± 8.4 (n = 10)	2.6 ± 5.9 (n = 13)	3.1 ± 8.2 (n = 18)
	p-value	**0.012**^a^	**<0.001**^a^	**<0.001**^a^
BPDAI Mucosal Score, mean ± SD	Pre	4.9 ± 3.1 (n = 6)	3.5 ± 3.7 (n = 8)	4.1 ± 2.5 (n = 13)
	Post	10.6 ± 8.5 (n = 6)	0.9 ± 1.2 (n = 7)	0.9 ± 1.7 (n = 8)
	p-value	0.062	**0.016**^a^	**0.008**^a^
Itching NRS Score, mean ± SD	Pre	6.3 ± 1.4	6.5 ± 2.9	6.4 ± 2.4
	Post	1.2 ± 1.6 (n = 10)	0.2 ± 0.6 (n = 13)	0.9 ± 1.9 (n = 18)
	p-value	**0.002**^a^	**<0.001**^a^	**<0.001**^a^

Dupi, Dupilumab; CS, Corticosteroids; pre, Pre-treatment; post, Post-treatment; BMZ, Basement Membrane Zone; DIF, Direct Immunofluorescence; IIF, Indirect Immunofluorescence using normal human skin; ssIIF, IIF using 1 M-NaCl-split normal human skin; BPDAI, Bullous Pemphigoid Disease Area Index; NRS, Numeric Rating Scale. IgG antibodies against BP180 NC16A, BP230, Desmoglein 1 (Dsg1) and Dsg3 were measured by MBL, Nagoya, Japan according to the manufacturer' instruction and the cut-off values in serum samples were 9 U/mL, 9 U/mL, 20 U/mL and 20 U/mL, respectively. The blood eosinophil count and percentage were measured using the coulter cellular analysis system (UniCel DxH 600, Beckman Coulter, California, USA) and the cut-off values were 0.02∼0.52*10^9^/L and 0.4∼8.0%, respectively. The level of serum total IgE was performed by Immuno CAP (Phadia/Thermofisher, Uppsala, Sweden) and the cut-off value in serum samples was 60 KU/L. p-value, post-treatment vs. pre-treatment. Statistical analyses were performed with Wilcoxon signed rank test.

**Bold**, p < 0.05 and the difference of corresponding indicators before and after treatment was significant.

a Post-treatment < pre-treatment.

For immunofluorescence detection in Dupi, Dupi + CS, and CS groups, the positive rates of DIF for linear IgG deposition to BMZ were 40.0% (2 of 5 cases), 73.3% (11 of 15 cases) and 71.4% (15 of 21 cases), respectively; the positive rates of DIF for linear C3c deposition to BMZ were 40.0% (2 of 5 cases), 60.0% (9 of 15 cases) and 81.0% (17 of 21 cases), respectively; the positive rates of ssIIF for IgG reactivity with epidermal side were 66.7% (8 of 12 cases), 76.2% (16 of 21 cases) and 83.3% (25 of 30 cases), respectively (Table 1).

By ELISA, all the serum samples (69 cases) of BP patients in the three groups were positive for anti-BP180 and/or anti-BP230 autoantibodies (IgG) at baseline. The data about serum total IgE level, blood eosinophil count and eosinophil percentage in BP patients of the three groups were also collected (Table 1).

The BPDAI was used to measure the disease activities of BP. The mean of BPDAI Total Score in the three groups at baseline were 48.1, 60.1 and 51.1, respectively (Table 1). In addition to BPDAI Total Score, other scores including BPDAI Erosion/Blister Score, BPDAI Urticaria/Erythema Score, BPDAI Mucosal Score and Itching NRS Score were also summarized in Table 1. In the present study, there were 6 of 13 (46.2%), 8 of 24 (33.3%) and 13 of 32 (40.6%) patients with oral mucosa lesions in Dupi, Dupi + CS and CS groups, respectively; and the mean of BPDAI Mucosal score at baseline were 4.9, 3.5 and 4.1, respectively (Table 1). Among these patients, 0% (0 of 5 cases), 42.9% (3 of 7 cases) and 25% (3 of 12 cases) were positive for herpes simplex virus type 1 by oral mucosal virus detection, respectively. These patients also underwent fungal detection on the erosive surface of the oral mucosa, but all were negative.

For Dupi + CS and CS groups, the median dose of oral prednisone used at baseline was 40 mg (range: 10–75 mg) and 50 mg (range: 20–80 mg), respectively; the median cumulative dosage of prednisone at the time of disease control were 225 mg (range: 30–675 mg) and 280 mg (range: 90–960 mg), respectively; and the median period of time to initiate the tapering were 7 days (range: 5–28 days) and 14 days (range: 7–28 days), respectively.

### Clinical and laboratory differences between pre-treatment and post-treatment in three groups of BP patients

Clinical and laboratory differences between pre-treatment and post-treatment in the three groups of BP patients were summarized in Table 1.

**Fig. 1:**
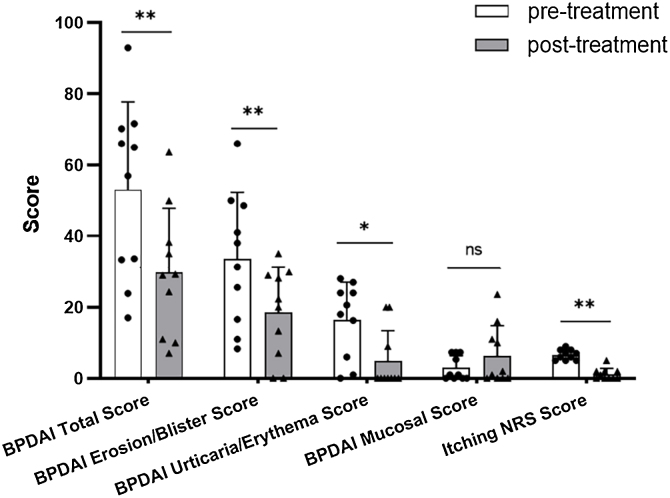
Comparison of clinical parameters before and after 2‒4 weeks’ treatment in Dupi group of BP patients. Five clinical scores for evaluation of BP patients. Statistical analyses were performed with Wilcoxon signed rank test. *p < 0.05; **p < 0.01; ns, not significant.

In Dupi group, the BPDAI Total (p < 0.01), Erosion/Blister (p < 0.01), Urticaria/Erythema (p < 0.05) and Itching NRS (p < 0.01) scores were all reduced significantly after 2–4 weeks’ treatment, but there was no significant decrease in the BPDAI Mucosal Score after 2–4 weeks’ treatment (Table 1 and Fig. 1). The mean serum total IgE level was elevated from 1246.6 KU/L at baseline to 1710.2 KU/L after 2–3 weeks’ treatment (n = 5). The mean serum levels of anti-BP180 (n = 4) and anti-BP230 (n = 3) autoantibodies (IgG) were changed from 85.5 U/mL and 8.8 U/mL at baseline to 82.1 U/mL and 31.7 U/mL after 1–3 months’ treatment, respectively. The blood eosinophil count and percentage were not reduced significantly after 2–3 weeks of treatment (Table 1).

In the Dupi + CS group, all the clinical parameters including the BPDAI Total (p < 0.001), Erosion/Blister (p < 0.001), Urticaria/Erythema (p < 0.001), Mucosal (p < 0.05) and Itching NRS (p < 0.001) scores were reduced significantly after 2–4 weeks’ treatment (Table 1). The serum level of anti-BP180 autoantibodies (IgG) was reduced significantly (p < 0.01) after 1–3 months treatment (Table 1). The blood eosinophil count and percentage were reduced significantly (both p < 0.001) after 2–3 weeks treatment (Table 1). However, there was no significant decrease in the serum total IgE level after 2–3 weeks’ treatment; and no significant decrease in the serum anti-BP230 autoantibodies (IgG) level was observed after 1–3 months’ of treatment (Table 1).

In the CS group, all the clinical parameters including the BPDAI Total (p < 0.001), Erosion/Blister (p < 0.001), Urticaria/Erythema (p < 0.001), Mucosal (p < 0.01) and Itching NRS (p < 0.001) scores were reduced significantly after 2–4 weeks’ treatment (Table 1). Both the serum levels of anti-BP180 (p < 0.001) and anti-BP230 (p < 0.01) autoantibodies (IgG) were reduced significantly after 1–3 months’ of treatment (Table 1). The blood eosinophil count and percentage were reduced significantly (both p < 0.001) after 2–3 weeks treatment (Table 1). But no significant decrease in the serum total IgE level was observed after 2–3 weeks’ of treatment (Table 1).

### Comparison of clinical and laboratory characteristics among the three groups of BP patients

**Fig. 2:**
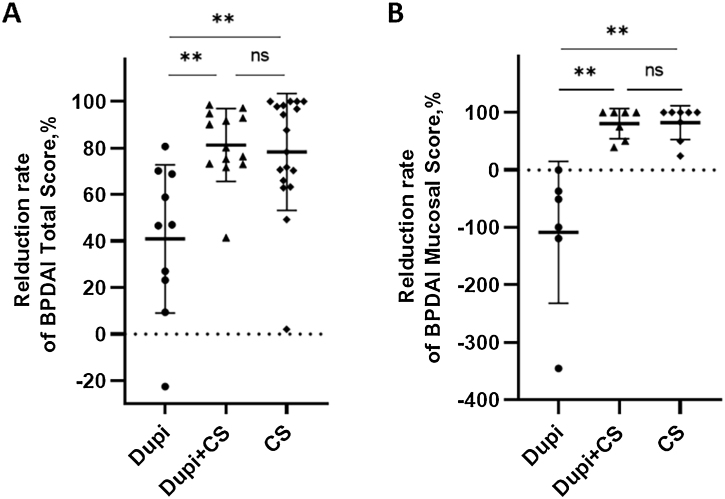
Comparison of reduction rates of two clinical parameters after 2‒4 weeks’ corresponding treatments among Dupi, Dupi + CS and CS groups. (A–E) Reduction rates of BPDAI Total Score (A), and BPDAI Mucosal score (B). Statistical analyses were performed with Steel-Dwass test for post-hoc pairwise comparisons between three groups after significant Kruskal-Wallis test. **p < 0.01; ns, not significant.

**Fig. 3:**
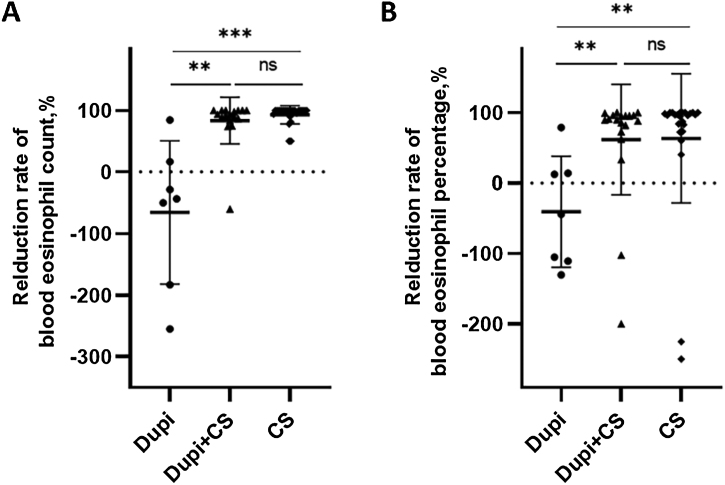
Comparison of reduction rates of blood eosinophil level after corresponding treatments among Dupi, Dupi + CS and CS groups. (A–B) Reduction rates of blood eosinophil count (UniCel DxH 600, Beckman Coulter, California, USA, 0.02∼0.52*10^9^/L) after 2–3 weeks’ treatment (A), and blood eosinophil percentage (UniCel DxH 600, Beckman Coulter, California, USA, 0.4∼8.0%) after 2–3 weeks’ treatment (B). Statistical analyses were performed with Steel-Dwass test for post-hoc pairwise comparisons between three groups after significant Kruskal-Wallis test. **p < 0.01; ***p < 0.001; ns, not significant.

Difference analyses of the reduction rates of clinical and laboratory characteristics after corresponding treatment among the three groups of BP patients were summarized in Fig. 2, Fig. 3, S2, and S3.

For clinical indicators, the reduction rates of both the BPDAI Total and Mucosal scores in Dupi group were significantly lower than those in Dupi + CS (both p < 0.01) and CS (both p < 0.01) groups, respectively, with no significant difference between the latter two groups (Fig. 2A and B). There were no significant differences in the reduction rates of BPDAI Erosion/Blister, Erythema, and Itching NRS scores among the three groups (Figs. S1‒S2).

For laboratory parameters, the reduction rates of the blood eosinophil count and percentage in the Dupi group were significantly lower than those in Dupi + CS (both p < 0.01) and CS (p < 0.001, p < 0.01) groups, respectively, with no significant difference between the latter two groups (Fig. 3A and B). However, there were no significant differences in the reduction rates of serum levels of total IgE, anti-BP180, and anti-BP230 autoantibodies (IgG) among the three groups (Figs. S3‒S4).

**Fig. 4:**
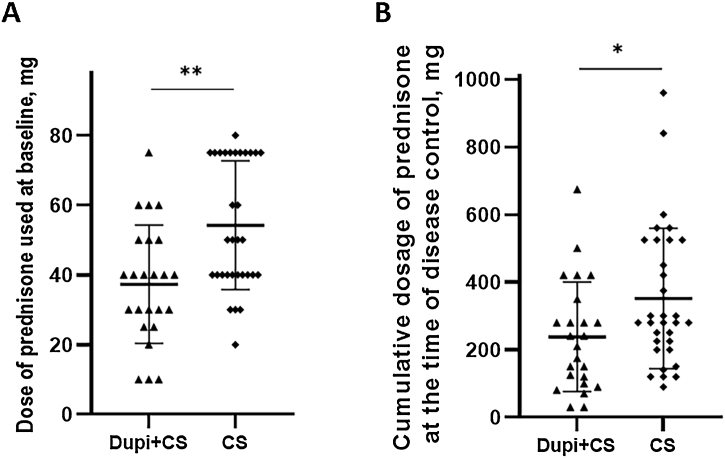
Comparison of dosage of prednisone administered between Dupi + CS and CS groups. (A) Dose of prednisone used at baseline. (B) Cumulative dosage of prednisone at the time of disease control. Statistical analyses were performed with Mann-Whitney *U* test and fisher’s exact test. *p < 0.05; **p < 0.01.

In the Dupi group, only 5 of 13 cases achieved disease control and the mean disease control period was 14.8 days (9–21 days); among them, 2 patients achieved complete remission with medicine at week 8. There were no significant differences in the disease control period, percentage of patients achieving complete remission during tapering at week 8, and relapse rates within one year between Dupi + CS and CS groups (Figs. S5‒S6). However, the dose of prednisone at baseline (37.3 ± 16.9 mg vs. 54.2 ± 18.4 mg, p < 0.01) and cumulative dosage of prednisone at the time of disease control (238.1 ± 162.3 mg vs. 351.7 ± 207.9 mg, p < 0.05) administered in Dupi + CS group were significantly less than those in CS group, respectively (Fig. 4A and B).

### Safety

As summarized in Table 2, a total of 23 adverse events occurred during the follow-up period. Four patients experienced transient peripheral blood eosinophilia, including three (23.1%, 3 of 13 cases) in Dupi group and one (4.2%, 1 of 24 cases) in Dupi + CS group. One patient (4.2%, 1 of 24 cases) in Dupi + CS group experienced conjunctivitis after two dupilumab injections. Compared with patients in the CS group (15.6%, 5 of 32 cases), patients in Dupi + CS group (8.3%, 2 of 24 cases) showed a decreased overall risk of infections (including serious or opportunistic infections). More cases of osteoporosis (6.3%, 2 of 32 cases), hypertension (9.4%, 3 of 32 cases), diabetes (6.3%, 2 of 32 cases) and hypodynamia (9.4%, 3 of 32 cases) were seen in the CS group. No deaths occurred during the follow-up period. No patients in the Dupi and Dupi + CS groups were discontinued due to adverse events.

**Table 2:** Summary of adverse events.

	Dupi group (n = 13)	Dupi + CS group (n = 24)	CS group (n = 32)
Eosinophilia, n (%)	3 (23.1%)	1 (4.2%)	0
Conjunctivitis, n (%)	0	1 (4.2%)	0
Infection, n (%)	0	2 (8.3%)	5 (15.6%)
Osteoporosis, n (%)	0	1 (4.2%)	2 (6.3%)
Hypertension, n (%)	0	0	3 (9.4%)
Diabetes, n (%)	0	0	2 (6.3%)
Hypodynamia, n (%)	0	0	3 (9.4%)

Dupi, Dupilumab; CS, Corticosteroids.

## Discussion

In the present study, BP patients were treated by dupilumab monotherapy mainly for the following reasons: 69.2% (9 out of 13 cases) of BP patients chose to use dupilumab treatment due to poorly controlled underlying diseases (such as diabetes, kidney disease and osteoporosis); 23.1% (3 out of 13 cases) of BP patients refused to use oral corticosteroid therapy; and 7.7% (1 out of 13 cases) of BP patients had a poor response to traditional corticosteroid therapy.

By the comparison of various clinical parameters before and after treatment, although the number of cases in Dupi group was relatively small, it can be inferred that dupilumab can improve the skin lesions and pruritus symptoms of BP, but it may be ineffective for the treatment of oral mucosal lesions (Fig. 1A and S7‒S8). Regarding this issue, there are a few points to consider: i) There is growing evidence that antibody-producing B-cells, autoreactive Th1 and Th2 mixed profiles, the complement system, and certain inflammatory factors are involved in the pathogenesis of BP,^34^, ^35^, ^36^, ^37^ and dupilumab can effectively inhibit Th2 inflammatory response, but it may have little effect on other immune and inflammatory pathways, which may be the reason why dupilumab monotherapy cannot completely control BP; (ii) There are currently no cases where dupilumab is ineffective or effective in treating mucosal damage in other diseases; (iii) Although the authors have ruled out the possibility of combined pemphigus, the authors have not conducted tests for mucous membrane pemphigoid (MMP)-related autoantibodies, which means the authors have not ruled out the possibility that the BP patients may be combined with MMP, and there is currently no literature on dupilumab in the treatment of MMP. Therefore, the reason why dupilumab is ineffective in treating BP oral mucosal lesions is still unclear, and further investigation is needed.

In the present study, most BP patients in Dupi + CS group were not suitable for long-term use of large-dose corticosteroids due to underlying diseases, but medium/low-dose corticosteroid treatment could not quickly control the disease. The conditions of these patients were rapidly controlled after adding to dupilumab treatment, which can achieve the effect of sufficient corticosteroid therapy. However, compared with the CS group, Dupi + CS group had less baseline dose and cumulative dosage of prednisone at the time of disease control and fewer adverse effects were reported. Two previous studies found that the disease control period of BP patients treated with dupilumab combined with corticosteroids was significantly shorter than that of BP patients treated with corticosteroids monotherapy, and the reduction rates of both the BPDAI Total Score and Itching NRS Score were significantly higher in the combined therapy than those of the corticosteroids monotherapy after 2 weeks’ treatment.^25^, ^28^ Therefore, both the results of the literature and the present study suggested that a combination of dupilumab and medium or low doses of corticosteroids might be superior to sufficient corticosteroid therapy.

In the present study, the serum anti-BP180 autoantibodies (IgG) level was significantly reduced in Dupi + CS and CS groups after 1–3 months’ treatment, which was consistent with a previous study that the serum anti-BP180 autoantibodies (IgG) levels in Dupi + CS and CS groups were both significantly reduced after 4-weeks’ treatment.^28^ These results indicated that both the corticosteroids monotherapy and its combination of dupilumab can significantly suppress the production of anti-BP180 autoantibodies (IgG) in BP patients. For the serum level of anti-BP230 autoantibodies (IgG), it was reduced significantly in the CS group after 1–3 months’ treatment, but not in the Dupi + CS group in the present study. However, Yang J et al. found that there was no significant change in the serum anti-BP230 autoantibodies (IgG) level both in the Dupi + CS and CS groups after 4 weeks’ treatment.^28^ The authors considered that the different conclusions may be caused by different follow-up periods between the present and previous studies. Although the sample data was relatively small in the Dupi group, the authors can infer that dupilumab monotherapy cannot significantly suppress the production of anti-BP180 and anti-BP230 autoantibodies (IgG) in BP patients.

Most studies have reported that higher serum IgE autoantibodies level is associated with more severe clinical manifestations of BP.^38^ Moreover, the serum total IgE level of BP patients in the remission stage was significantly lower than that in an advanced stage.^39^ In the present study, the serum total IgE level in the Dupi group was elevated after 2–3 weeks treatment, although no significant statistical differences were observed due to relatively small sample data (n = 5). However, the serum total IgE levels in Dupi + CS and CS groups were not changed significantly after 2–3 weeks treatment. Yang J et al. found that the serum total IgE levels were reduced significantly in the Dupi + CS group after 4 weeks’ of treatment and no significant change was observed in the CS group.^28^ The authors considered that the different conclusions may be caused by different follow-up periods of the CS group between the present and previous studies.

Studies have found that the level of eosinophils is elevated in the peripheral blood of over 50% of untreated patients with BP, which has been proven to be positively correlated with both disease and itch severity.^40^ The results of the present study indicated that both the corticosteroid monotherapy and its combination of dupilumab can significantly reduce the blood eosinophil level of BP patients after 2–3 weeks’ treatment, but dupilumab monotherapy cannot significantly reduce the blood eosinophil level.

The data in the present study, especially the comparison results of treatment effects and adverse effects of the three groups, implied that: i) For patients who have only skin lesions and are not suitable for traditional corticosteroids and immunosuppressive therapy due to underlying diseases, dupilumab monotherapy may be considered; and its advantage is safe, and may not aggravate the development of underlying diseases, and disadvantage is expensive and most patients may get better after treatment but may not achieve complete control of the disease; (ii) For patients who are ineffective with traditional treatments or unsuitable for large dose of corticosteroids due to underlying diseases and unable to achieve disease control by medium or low doses of corticosteroids, dupilumab combined with corticosteroids therapy could be considered; the advantage of this therapeutic strategy is that most patients can rapidly control the disease and achieve complete remission; the disadvantage is expensive for long-term use.

## Conclusion

In conclusion, this study provides real-world practice evidence that dupilumab monotherapy based on the regimen for the treatment of atopic dermatitis can significantly improve the skin lesions and pruritus symptoms of BP, but it may be ineffective for the treatment of oral mucosal lesions in BP patients. A combination of dupilumab and medium or low doses of corticosteroids could control BP rapidly and safely and exhibits superior safety in comparison with sufficient corticosteroids therapy. The findings of the present study provide useful guidance on the clinical application of dupilumab in the treatment of BP patients, especially for patients who are resistant to corticosteroids therapy or are not suitable for large-dose corticosteroids therapy.

## Financial support

This work was supported by the Scientific Research Project of Jiangsu Provincial Health Commission (ZD2021035), National Key R&D Program of China (2022YFC3601800) and Education Fund Item of Liaoning Province (LJKMZ20221843).

## Authors’ contributions

Guirong Liang: Data collection, analysis and interpretation; statistical analysis; Writing of the manuscript or critical review of important intellectual content; Intellectual participation in the propaedeutic and/or therapeutic conduct of the studied cases; Critical review of the literature; Final approval of the final version of the manuscript.

Hua Qian: Effective participation in the research guidance.

Chao Sun: Data collection, or analysis and interpretation of data.

Hanmei Zhang: Data collection, or analysis and interpretation of data.

Zhiliang Li: Intellectual participation in the propaedeutic and/or therapeutic conduct of the studied cases.

Suo Li: Intellectual participation in the propaedeutic and/or therapeutic conduct of the studied cases.

Ke Jing: Intellectual participation in the propaedeutic and/or therapeutic conduct of the studied cases.

Chenjing Zhao: Intellectual participation in the propaedeutic and/or therapeutic conduct of the studied cases.

Yuan Wang: Intellectual participation in the propaedeutic and/or therapeutic conduct of the studied cases.

Ruiyu Xiang: Intellectual participation in the propaedeutic and/or therapeutic conduct of the studied cases.

Xiaoguang Li: The study concept and design; Effective participation in the research guidance.

Suying Feng: The study concept and design; Effective participation in the research guidance; Intellectual participation in the propaedeutic and/or therapeutic conduct of the studied cases.

## Conflicts of interest

None declared.
