# Supplementary material for: Dupilumab, corticosteroids and their combination for the treatment of bullous pemphigoid
Source: An Bras Dermatol. 2024 Dec 17;100(2):243–52. doi: 10.1016/j.abd.2024.04.012 (PMC11962819; doi:10.1016/j.abd.2024.04.012)
Supplement: Supplementary file 1 [file mmc1.docx]

ABD-D-24-00159_Supplementary Material

**Figure S1 Comparison of reduction rates of clinical parameters after 2‒4 weeks’ corresponding treatments among Dupi, Dupi+CS and CS groups.** (A‒B) Reduction rates of BPDAI Erosion/Blister score (A), and BPDAI Urticaria/Erythema score (B). Statistical analyses were performed with Steel-Dwass test for post-hoc pairwise comparisons between three groups after significant Kruskal-Wallis test. ns, not significant.

**Figure S2 Comparison of reduction rate of Itching NRS score after 2‒4 weeks’ corresponding treatments among Dupi, Dupi+CS and CS groups.** Statistical analyses were performed with Steel-Dwass test for post-hoc pairwise comparisons between three groups after significant Kruskal-Wallis test. ns, not significant.Figure S3

**Figure S3 Comparison of reduction rates of laboratory parameters after corresponding treatments among Dupi, Dupi+CS and CS groups.** (A‒B) Reduction rates of serum anti-BP180 autoantibodies (IgG) level (MBL, Nagoya, Japan, <9 U/mL) (A), and serum anti-BP230 autoantibodies (IgG) level (MBL, Nagoya, Japan, <9 U/mL) (B) after 1‒3 months’ treatment. Statistical analyses were performed with Steel-Dwass test for post-hoc pairwise comparisons between three groups after significant Kruskal-Wallis test. ns, not significant.

**Figure S4 Comparison of reduction rate of serum total IgE level (Phadia/Thermofisher, Uppsala, Sweden, < 60 KU/L) after 2‒3 weeks’ corresponding treatments among Dupi, Dupi+CS and CS groups.** Statistical analyses were performed with Steel-Dwass test for post-hoc pairwise comparisons between three groups after significant Kruskal-Wallis test. ns, not significant.

**Figure S5 Comparison of treatment effects between Dupi+CS and CS groups.** (A) Disease control period. (B) Percentage of patients achieving complete remission during tapering at week 8. Statistical analyses were performed with Mann-Whitney *U* test and fisher’s exact test. ns, not significant.

**Figure S6 Comparison of relapse rates within one year of treatment between Dupi+CS and CS groups.** Statistical analyses were performed with fisher’s exact test. ns, not significant.

**Figure S7 Comparison of skin lesions before and after 4-weeks’ treatment in one BP patient of Dupi group.** A showed the characteristics of admission, while B showed the characteristics after 4-weeks’ treatment with dupilumab.

**Figure S8 Comparison of oral mucosa lesions before and after 4-weeks’ treatment in one BP patient of Dupi group.** A showed the characteristics of admission, while B showed the characteristics after 4-weeks’ treatment with dupilumab.
